# An earphone wire inside the urinary bladder: A case report and comprehensive literature review of genitourinary polyembolokoilamania

**DOI:** 10.1016/j.radcr.2022.01.080

**Published:** 2022-03-02

**Authors:** Haviv Muris Saputra, Yudhistira Pradnyan Kloping, Johan Renaldo, Lukman Hakim

**Affiliations:** a Department of Urology, Faculty of Medicine, Universitas Airlangga, Surabaya, East Java, Indonesia; b Dr Soetomo General-Academic Hospital, Surabaya, East Java, Indonesia; c Rumah Sakit Universitas Airlangga Teaching Hospital, Surabaya, East Java, Indonesia

**Keywords:** Genitourinary foreign body, Urethral foreign body, Bladder foreign body, Polyembolokoilamania

## Abstract

Self-inserted urinary bladder foreign bodies for sexual gratification generate a significant challenge for physicians due to its difficult diagnosis and management. Most patients were late to be admitted due to embarrassment leading to serious short-term and long-term complications. We report a 34-year-old male with an earphone wire as a urinary bladder foreign body. The findings in the patient were compared with the currently published reports through a comprehensive literature review to evaluate the current strategy for diagnosis and management for self-inserted genitourinary foreign bodies to achieve sexual pleasure.

## Introduction

A genitourinary foreign body represents a rare finding, even though the number of cases has risen in the past few decades. However, among all genitourinary organs, the urethra, and the bladder is the most common site for a foreign body [1]. Urologists have been facing this issue for years since it possesses a significant challenge based on its diagnosis and management [2]. The etiopathogenesis of the urinary bladder foreign body often involves self-insertion, iatrogenic process, or migration from other adjacent organs. Self-insertion motivated by sexual gratification is the most common cause, which could happen in non–psychiatric patients with certain fetishes [3]. However, several non–psychotic patients are also present with this sexual deviation, termed polyembolokoilamania [4]. A variety of objects, such as pencils, thermometers, electric cables, wires, etc. may be inserted [5]. Unexpected organic objects like olive seeds, and kidney beans could also be inserted [5,6]. Performing a complete initial assessment of the patient via history taking may be difficult as some patients feel embarrassed or guilty to seek immediate medical attention. Most patients are late to be admitted, thus severe local or systemic complications may have already taken place. Common complaints of these patients include dysuria, urinary retention, lower abdominal pain, hematuria, urethral discharge, and fever [7]. Smaller, less impacted objects may cause persisting chronic manifestations like recurrent urinary tract infections (UTI). In some cases, urinary stones and urosepsis may develop [8]. However, unexpected cases may also occur by accident in patients due to iatrogenic causes or self-inflicted [9]. Thus, a thorough and detailed history taking and physical examination, followed by proper imaging modalities are necessary to successfully manage these patients. In Indonesia, these cases are rarely reported due to their taboo nature, causing difficulties in properly managing the problem among physicians. Therefore, we report a 34-year-old male with an earphone wire in his bladder. The case was compared with the currently published reports through a comprehensive literature review to evaluate the current strategy for diagnosis and management for genitourinary foreign bodies.

## Case presentation

A 34-year-old male was admitted to the emergency department of Dr Soetomo General-Academic Hospital with a chief complaint of lower abdominal pain during urination for 3 days. The pain was felt from the start until the end of urination. Terminal hematuria was also reported by the patient. Fever, nausea, and vomiting were denied by the patient. The patient admitted to having inserted an earphone wire into his urethra. He had done this often for 3-5 times a week while masturbating. We consulted the patient to the psychiatric department and the psychiatrist concluded that the patient had no psychotic symptoms, obsessive-compulsive disorder, anxiety disorder, or depression. The patient's action was performed based on sexual pleasure and gratification. The patient also claimed to be having financial and family problems, however, the association was unlikely. The behavior became a problem when the patient could not take the wire out as it was lodged in the bladder. Physical examination showed suprapubic tenderness. Urinalysis showed a high red blood cell and white blood cells count.

### Investigations/Imaging findings

**Fig. 1:**
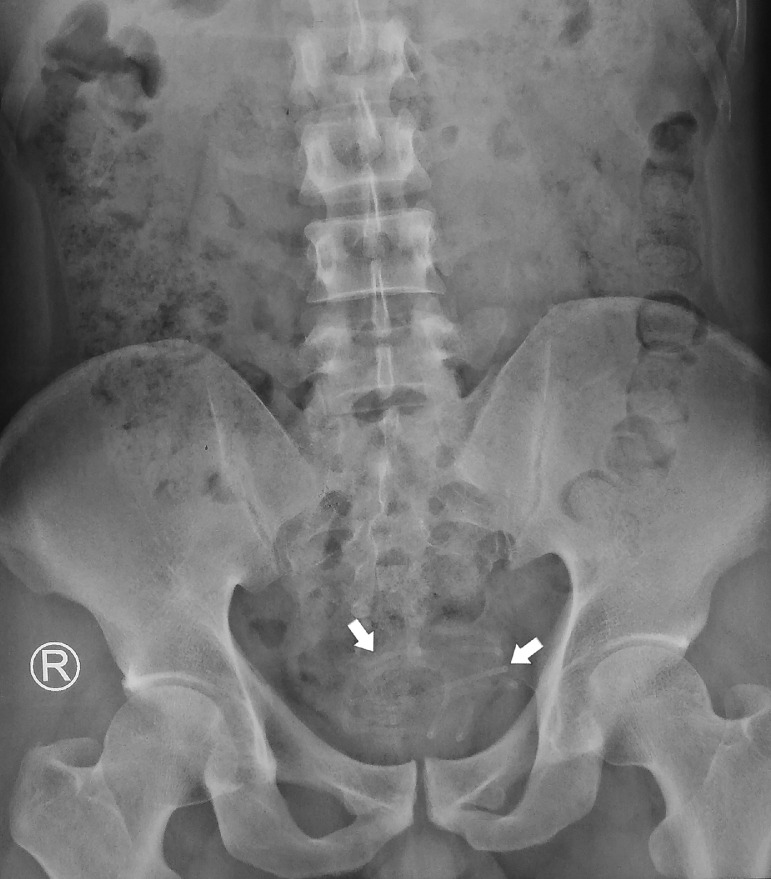
Plain pelvic X-Ray showing a semi-radio-opaque tubular shadow in the pelvis, as indicated by the arrows.

**Fig. 2:**
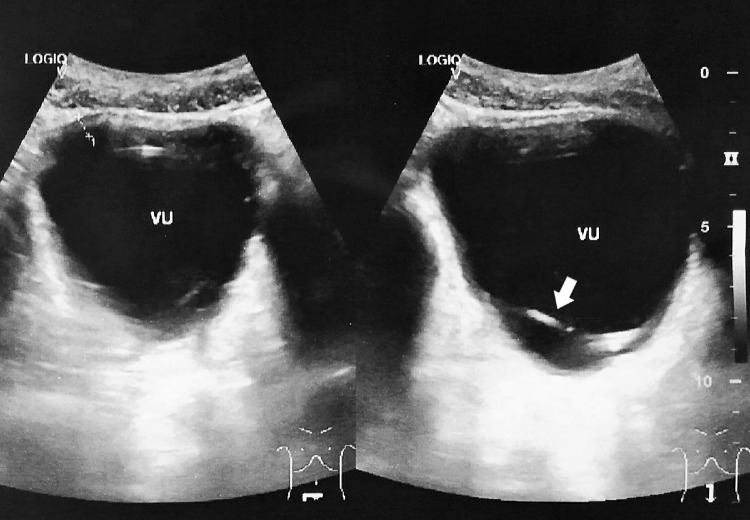
USG examination of the bladder showing a hyperechoic shadow, indicating a foreign body, as shown by the arrows.

**Fig. 3:**
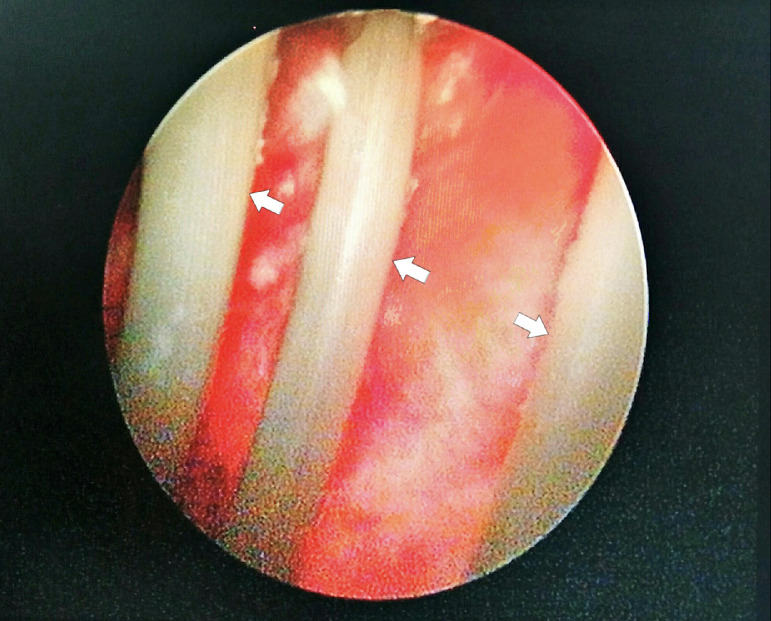
Cystoscopy examination showed a tubular object as shown by the arrows and hyperemia of the bladder mucosa.

Pelvic plain radiographic X-Ray showed a semi-radiopaque shadow, suggesting a possible foreign body in the pelvic cavity, as shown in Fig. 1. An ultrasonography (USG) examination also suggested a foreign body in the bladder, as shown in Fig. 2. We performed a cystoscopy under general anesthesia. During the procedure in Fig. 3, hyperemia of the bladder wall could be seen. A urothelial mass or encrustation was not seen. The wire was visible and quickly identified. It was coiled and fortunately was not attached to the bladder wall.

### Differential diagnosis

The patient was diagnosed with a urinary bladder foreign body based on history taking and imaging results.

### Treatment

**Fig. 4:**
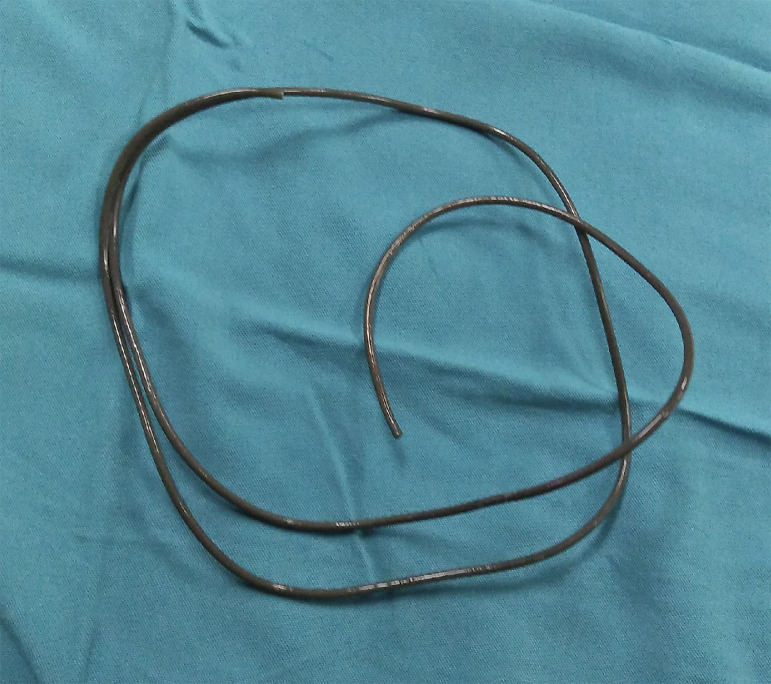
The extracted earphone wire, 80 cm in length, and 2-3 mm in diameter.

Extraction was performed using grasping forceps. An earphone wire, 2-3 mm in size and 80 cm in length was extracted, as shown in Fig. 4.

### Outcome and follow-up

The patient was discharged on the second day without any bladder residue. There were no complaints of postoperative lower urinary tract symptoms. The patient was later referred to the psychiatric department again and was diagnosed with polyembolokoilamania. He showed no apparent psychotic behaviors and was mentally well.

## Discussion and literature review

**Fig. 5:**
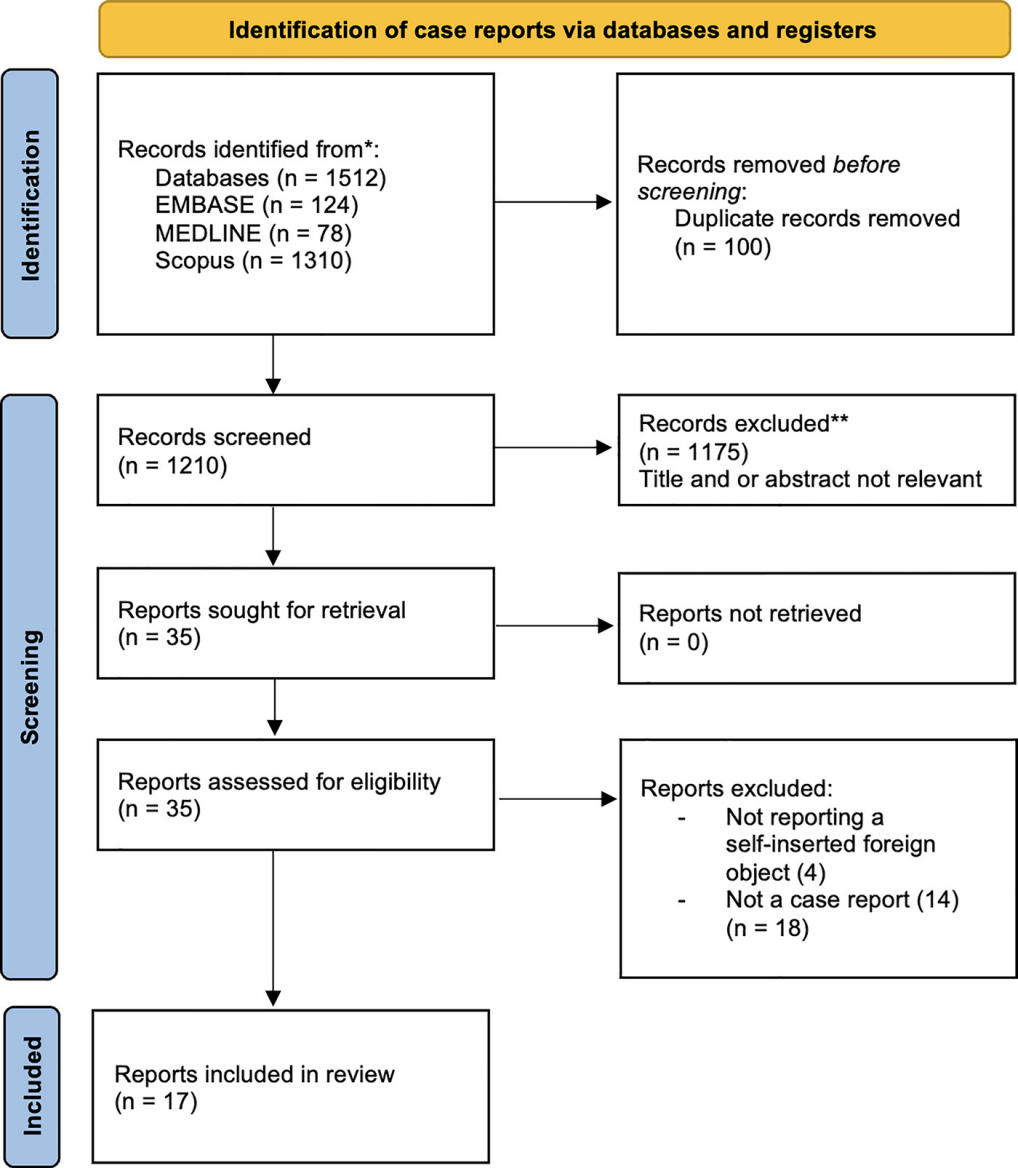
Systematic primary and secondary screening based on the PRISMA flowchart.

Since it was first reported, numerous cases of bladder foreign bodies of various shapes, and forms have been reported [10]. It represents a specific entity occurring mostly in the context of psycho-affective disorders. As what was found in this patient, even though he was mentally sound, the voluntary introduction of objects into the urethral meatus for sexual gratification reflects a psychopathological condition [4]. We have performed a systematic search in the Embase, Medline, and Scopus databases for previous similar case reports based on the Preferred Reporting Items for Systematic Reviews and Meta-Analyses (PRISMA) guideline using relevant keywords related to self-inserted genitourinary foreign bodies [11]. A total of 1512 articles were obtained in the initial search. After the primary and secondary screenings were conducted as shown in Fig. 5, we obtained a total of 17 relevant case reports reporting self-insertion of foreign bodies into the urethra and bladder [3,^4^,^6^,[12], [13], [14], [15], [16], [17], [18], [19], [20], [21], [22], [23], [24], [25]. The details and characteristics of each report are shown in Table 1. Most patients are male, indicating a possible predominance of male patients with self-insertion autoeroticism or a higher tendency to seek help compared to female patients. However, sex predilection for this fetish requires further investigation through epidemiologic studies. The objects reported varied from inorganic to organic objects. Two of the most interesting objects reported consist of 4 kidney beans and an object resembling a worm with an encrustation [6,12]. Most reports consist of tubular objects, including cables similar to the earphone wire in this case report. When a wire is inserted into the urethra, the terminal part may be stuck in the bladder with a portion of the wire remaining in the urethra. This becomes a problem when the bladder end forms a loop or is knotted during bladder contraction, preventing self-retrieval [14]. The mechanism of insertion and complex shape of the object may cause immediate or delayed complications. The signs and symptoms felt by the patients may be caused by the direct contact of the object with the bladder mucosa or complications arising from the long-term position of the object. Both acute and chronic complications, such as hematuria and urinary retention to vesicolithiasis and urosepsis have been previously reported [26], [27], [28]. In this review, we have discovered that the complications are related to the delay of treatment caused by the patients not immediately seeking help. The main concern involving patients with self-inserted foreign bodies is their late admittance due to embarrassment or guilt. Severe complications, such as stone formation, recurrent UTI, urinary retention, and necrotic tissue were reported [1,^12^,^15^,23]. More severe complications may occur due to dangerous corrosive substances like batteries as reported by Bedi et al [16]. A detailed history consisting of information regarding the nature of the foreign body is important to recommend a proper strategy. Creating and maintaining trust between the physician and the patient would allow the patient to be more open and honest. Confirming the presence, size, and the number of the objects can be performed using imaging modalities such as ultrasonography and plain X-Ray [29]. Current reports in this review suggest that USG and X-Ray are adequate for identifying most genitourinary objects. However, certain cases may require the use of a urethrogram or CT-Scan due to the material, shape, and location of the objects that may be difficult to visualize. Schmitt et al. initially thought that the foreign body in the patient was a parasitic worm, however, upon extraction, it was found to be an inorganic object mimicking the shape of a worm [12]. Thus, to fully visualize the object cystoscopy is necessary. The procedure is also used for management, by assisting the use of forceps or grasper to grab the object. Removal of the foreign body should always be performed with as minimal trauma as possible. Large objects may even require an open suprapubic cystostomy. In the reports found during the systematic search, most physicians attempted to use the endoscopic approach as the initial procedure. Difficulties in extraction without damaging the bladder and urethral mucosa due to the shape and location of the objects led to the consideration of using an open surgical approach [6,^22^,24]. In certain cases with severe complications, such as stone formation, vesicolithotomy might be necessary [12,15]. Extracting urethral foreign bodies might also require dorsal meatotomy instead of forcefully extracting the object [4,23]. Currently, new approaches to efficiently extract genitourinary foreign bodies, while preventing mucosal injury have been introduced. A report in 2021 introduced a novel technique of using an Endoloop to remove a bladder foreign body endoscopically for objects that are difficult to be extracted using a grasper or basket [30]. Many patients in the previous reports are diagnosed with mental illness or psychosis. However, there are mentally stable patients with unique particular fetishes, as shown in this report [31]. Nevertheless, these patients should be referred to the psychiatric department for assessment to prevent future recurrence.

**Table 1:** Case reports’ characteristics.

Author (Y)	Country	Age (Y)	Sex	Object	Location	Diagnostic Modality	Management	Complications
Trehan (2007)	UK	50	Male	Telephone cable wire	Urethra	X-Ray	Urethral extraction under local anesthesia	Urethral bleeding and incontinence
Naidu (2013)	Australia	70	Male	10 cm steel dining fork	Urethra	X-Ray, CT-Scan, urethrocystoscopy	Extraction under GA with lidocaine gel and Rampley forceps	Urethral bleeding and Hematuria
Jain (2018)	India	27	Male	4 kidney beans	Bladder	X-ray, USG, RUG, Micturating Cystourethrogram	Open suprapubic incision	Not reported
Raheem (2014)	Egypt	18	Female	Pen	Bladder	Urinalysis, USG, X-Ray, CT-Scan	Cystoscopy	Severe dysuria and
Imai (2011)	Japan	49	Male	140 cm vinyl tube	Bladder	USG and X-ray	Cystoscopy and open suprapubic incision	Hematuria
Moon (2010)	South Korea	50	Male	Round magnets, rod-shaped materials	Urethra and bladder	X-ray	Meatotomy and cystoscopy	Vesicolithiasis
		51	Male	5 cm green-colored tube	Urethra and bladder	RUG	Suprapubic cystostomy and external urethrotomy	Necrotic tissue
Ahmed (2016)	India	36	Male	Mobile charger cable and/or metallic wire	Bladder	USG and X-Ray	Cystoscopy and open suprapubic incision	Hematuria and urinary retention
Cam (2019)	Turkey	45	Male	Nail scissor	Urethra	X-Ray	Urethral extraction under local anesthesia	Urethral bleeding
Schmitt (2012)	USA	63	Male	16.0 × 1.3 cm non–organic FB resembling a worm with encrustation (90% ammonium urate and 10% uric acid crystals)	Bladder	USG and pelvic CT-Scan	Cystoscopy and vesicolithotomy	UTI, vesicolithiasis
Irekpita (2011)	Nigeria	34	Male	46 cm PVC coated electric wire	Bladder	USG and X-Ray	Suprapubic cystostomy	UTI
Chabouni (2022)	Tunisia	45	Female	Intravaginal foreign body and/or glass covered with urinary stone	Bladder	X-Ray	Suprapubic cystolithotomy under GA	Recurrent cystitis
Ogbetere (2021)	Nigeria	32	Male	Earphone cable	Urethra and bladder	X-Ray	Suprapubic cystostomy	Not reported
Elmortaji (2019)	Morocco	26	Male	Tip of pen	Bladder	USG and X-Ray	Cystoscopy followed by surgical extraction	Urethritis
		24	Male	12 cm pen	Urethra	X-Ray	Cystostomy	Traumatic urethral mucosa lesion
		80	Male	2 coins	Bladder	USG	Cystostomy	Vesicolithiasis, UTI
		62	Male	Condom	Bladder	USG	Cystoscopy	Urolithiasis
Bedi (2010)	UK	62	Male	2 triple A size battery	Bladder	X-Ray	Urethroscopy and cystoscopy	Necrosis and recurrent UTI
Winot (2021)	USA	25	Female	Lip gloss container	Bladder	X-Ray, CT-Scan	Cystoscopy	Urinary frequency, dysuria
Loufopoulos (2021)	UK	15	Male	Knotted USB Cable	Urethra	X-Ray and Fluoroscopic Urethrogram	Penoscrotal incision	Scarring due to urethral injury
Bonatsos (2021)	UK	70	Male	2 pens, 6 mm in diameter	Urethra	X-Ray	Urethroscopy	Penoscrotal swelling, voiding difficulties, cystitis

## Conclusion

A detailed assessment via history taking, physical examination, and imaging modalities based on a good rapport between the physician and patient is necessary to identify a genitourinary foreign body before suggesting a treatment recommendation. The principle of management consists of total removal and complete clearance of the object via cystoscopy. However, it may be replaced with a more invasive surgical approach, if warranted based on the foreign object's size, shape, and complex location.

## Patient consent and ethical approval

Informed consent was obtained for the publication of this case report and accompanying images. This report has been approved by the Dr Soetomo General-Academic Hospital ethical committee for research and publication (0725/LOE/301.4.2/XII/2021).

## Funding

No author received financial or material support for this report. No author has a financial or proprietary interest related to the report.

## Author contributions

HMS, YPK, JR, and LH contributed equally to this article. All authors have read the manuscript and agreed to the contents.
